# Human Papillomavirus and Survival of Sinonasal Squamous Cell Carcinoma Patients: A Systematic Review and Meta-Analysis

**DOI:** 10.3390/cancers13153677

**Published:** 2021-07-22

**Authors:** Anish Sharma, Alice L. Tang, Vinita Takiar, Trisha M. Wise-Draper, Scott M. Langevin

**Affiliations:** 1 Medical Sciences Program, University of Cincinnati College of Medicine, Cincinnati, OH 45267, USA; sharm2a5@mail.uc.edu; 2 Department of Otolaryngology, University of Cincinnati College of Medicine, Cincinnati, OH 45267, USA; tangac@uc.edu; 3 University of Cincinnati Cancer Center, Cincinnati, OH 45267, USA; takiarva@uc.edu (V.T.); wiseth@uc.edu (T.M.W.-D.); 4 Department of Radiation Oncology, University of Cincinnati College of Medicine, Cincinnati, OH 45267, USA; 5 Cincinnati VA Medical Center, Cincinnati, OH 45220, USA; 6 Department of Internal Medicine, Division of Hematology & Oncology, University of Cincinnati College of Medicine, Cincinnati, OH 45267, USA; 7 Department of Environmental & Public Health Sciences, Division of Epidemiology, University of Cincinnati College of Medicine, Cincinnati, OH 45267, USA

**Keywords:** HPV, cancer, sinus, paranasal, nasal cavity, overall survival, disease-free survival

## Abstract

**Simple Summary:**

Human papillomavirus (HPV) has been associated with multiple cancers in the anogenital and upper aerodigestive tracts. In the head and neck region, HPV-positive cancers are common in oropharynx, with rising incidence and a well-established association with more favorable patient outcomes. However, the relationship with prognosis of sinonasal squamous cell carcinoma (SNSCC) has been much less often studied and is presently unclear. To better elucidate this relationship, we performed a systematic review and meta-analysis of the biomedical literature to determine the aggregate effect across studies. In doing so, we observed significantly better overall survival associated with HPV-positive SNSCC. Therefore, we conclude that HPV testing may be useful for determining patient prognosis and potentially guiding treatment decisions.

**Abstract:**

Human papillomavirus (HPV) is detectable in a subset of sinonasal squamous cell carcinoma (SNSCC), but the impact on patient outcomes is presently unclear due to a modest number of studies with limited statistical power. Therefore, we conducted a systematic review and meta-analysis to better clarify this relationship. A PubMed search was conducted to identify all studies reporting on overall (OS) or disease-free survival (DFS) for SNSCC by HPV status. Hazard ratios (HR) and corresponding 95% confidence intervals (CI) were extracted or, when not provided, indirectly estimated from each manuscript. Summary survival curves for 5-year OS and estimating survival probability by HPV status at pre-specified time intervals from study-specific Kaplan-Meier curves generated 2-year DFS. Log HRs and log CIs were combined across studies to generate summary estimates and a corresponding 95% CIs for OS and DFS. We identified ten unique studies reporting on OS and four for DFS. We observed a significant association between HPV and OS (summary HR = 0.51, 95% CI: 0.38–0.70) with relatively low heterogeneity between studies. These results indicate that HPV is a significant predictor of more favorable survival for SNSCC, and thus may be a useful biomarker for prognostication and, potentially, treatment modulation.

## 1. Introduction

Sinonasal cancers are relatively rare malignancies that account for only 3–5% of all head and neck cancers in the United States, with an incidence rate of approximately 5.6 new cases per million population [1]. Sinonasal squamous cell carcinomas (SNSCC) comprise nearly half of these cancers [2]. Overall 5-year survival is estimated between 30–50% regardless of treatment, with local recurrence being the most common cause of mortality [3]. Due to the complex anatomy of the sinonasal tract and the proximity of surrounding organ systems, primary treatment methods, such as surgery and radiotherapy, result in high morbidity and complications [4].

Human papillomavirus (HPV) is well established as a major risk factor for oropharyngeal squamous cell carcinoma (OPSCC) for which it is associated with more favorable patient outcomes [5,6,7,8] and, as such, less aggressive treatment options are being explored for patients with HPV-positive tumors [9]. HPV is also an emerging risk factor for SNSCC, but the relationship with patient prognosis is presently much less clear. To date, studies reporting on the association of HPV with SNSCC survival have been predominately modest to small with limited statistical power and precision, largely owing to the relative rarity of these cancers, and have presented conflicting results. Studies have also used varying assays to detect HPV, including HPV DNA- and E6/E7 mRNA-based approaches, as there is currently no consensus gold standard approach for the detection of HPV in SNSCC. Further, while p16 protein expression works reasonably well as a surrogate marker for HPV in OPSCC [10], its reliability for SNSCC is suspect due to numerous studies reporting discordance with direct HPV analyses [11,12,13,14,15,16], which is similarly reflected in other non-oropharyngeal head and neck squamous cell carcinoma [17]. This is not wholly surprising, as p16 can become overexpressed due to factors beyond HPV infection, such as inactivation of Rb1 or other related genes regulating cell cycle [18].

The objective of this meta-analysis was to comprehensively assess the literature and generate summary estimates to better elucidate the relationship between HPV and overall (OS) and disease-free survival (DFS) among patients with SNSCC.

## 2. Results

### 2.1. Study Selection

**Figure 1:**
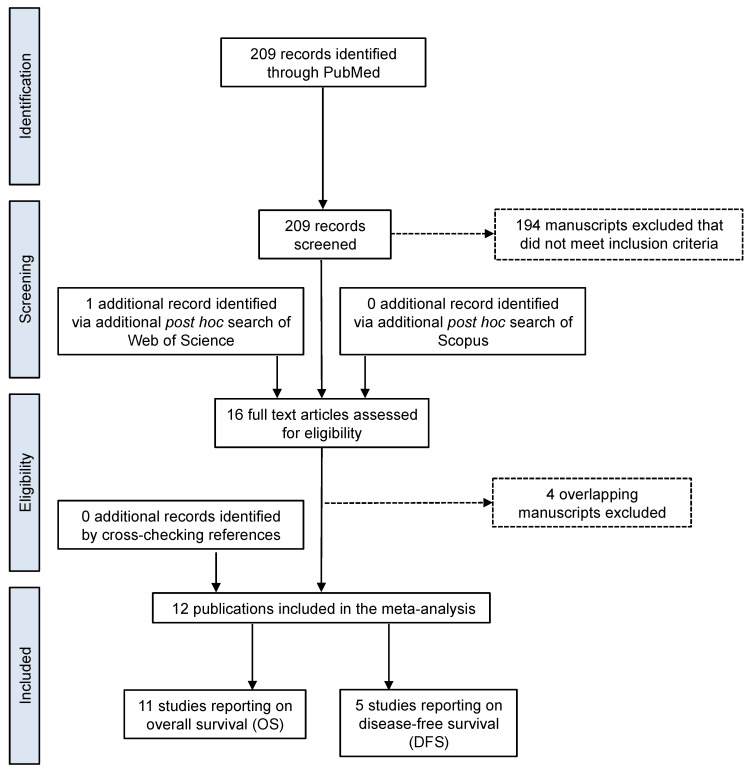
Flow diagram of the study identification and selection process for inclusion in the meta-analysis of overall and disease-free survival by HPV-status for patients with sinonasal squamous cell carcinoma.

A flow diagram of the study identification, screening, and inclusion process is presented in Figure 1. A comprehensive PubMed search for original research articles reporting data on the association of HPV and OS and/or DFS for SNSCC returned 209 potential manuscripts, of which 15 met the inclusion criteria [4,11,12,14,15,16,19,20,21,22,23,24,25,26], with 100% agreement between reviewers (AS and SML). One additional unique study [27] was identified via post hoc search of Web of Science; no additional studies were identified via post hoc search of Scopus or by crosschecking references. Three sets of overlapping study populations were identified among the 15 studies, including four studies that queried the National Cancer Database [4,23,24,26], two from the University of Barcelona Hospital Clinic [19,28], and two from the Kyushu Cancer Center [12,22]. In the case of the latter, both studies reported on OS, so the more inclusive was retained for the meta-analysis of OS [12], but only the less inclusive of the two reported on DFS [22], and therefore was also retained for the meta-analysis of DFS. In all, 11 unique studies reported on OS by HPV-status and combined for a total of 924 cases (252 HPV-positive (HPV+) and 672 HPV-negative (HPV-)) [11,12,14,15,16,19,20,21,24,25,28], while five eligible studies reported on DFS [14,19,22,25] with a combined total of 279 cases (54 HPV+ and 225 HPV-). HR was indirectly estimated for 7 of the 11 studies reporting on OS, since six did not provide any HR estimate [14,15,20,21,25,28] and one reported an HR but did not provide 95% confidence intervals or variance [12]; HR was also indirectly estimated for four of the five studies reporting on DFS [14,22,25,28]. One study [28] did not provide Kaplan-Meier survival plots, and therefore was not included in the summary survival curves for OS or DFS.

### 2.2. Study Characteristics

A description of the studies included in the meta-analyses is provided in Table 1. Studies were conducted in five different countries (USA, Japan, Spain, Slovakia, Czech Republic). The 11 studies that reported OS by HPV-status ranged in size from 22 SNSCC cases (Cohen et al. [14]) to 382 SNSCC cases (Oliver et al. [24]), with a median sample size of 49 cases; while the five studies that reported DFS by HPV-status ranged from 22 SNSCC cases (Cohen et al. [14]) to 101 SNSCC cases (Jiromaru et al. [22]), with a median sample size of 60 cases. The median frequency of HPV+ cases across studies was 29% (range: 9–62%). Subjects in the studies were mostly older adults and predominately male. The HPV detection method varied across studies, with six of the twelve included studies having used an HPV DNA-based approach [11,19,20,21,25,28], four studies having used assays for detection of high-risk HPV E6/E7 mRNA [12,14,16,22], and one having used a combination of HPV DNA and E6/E7 mRNA [15]; the study using the National Cancer Database did not specify an HPV testing format [24].

### 2.3. Qualitative Assessment of Study Quality and Risk of Bias

Qualitative scoring of the study quality and potential risk for bias is presented in Table 2. The majority of studies (n = 8) were scored as low quality/high risk for bias. This was largely driven by low sample size, including a small number of HPV+ SNSCC cases—which should not be surprising given the relatively rare nature of SNSCC—and failure to statistically adjust for potential confounding factors.

### 2.4. Meta-Analysis of Overall Survival

**Figure 2:**
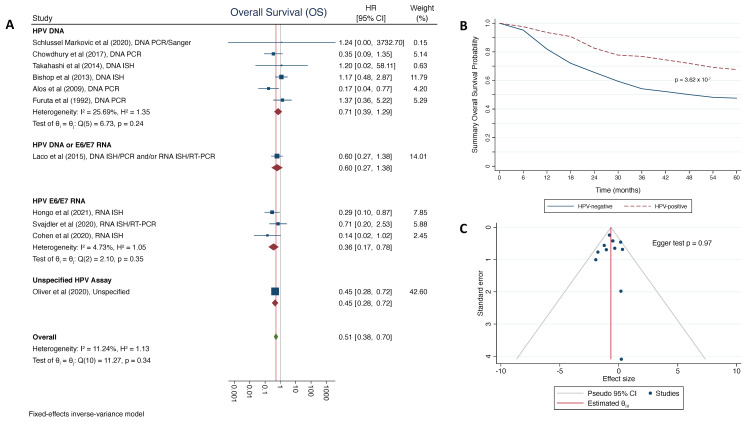
Summary estimates for the association between human papillomavirus (HPV)-status and overall survival (OS) for patients with sinonasal squamous cell carcinoma (SNSCC): (**A**) forest plot of overall survival meta-analysis with summary hazard ration (HR) stratified by HPV detection method; (**B**) summary Kaplan-Meier curve for 5-year OS of SNSCC patients by HPV status with log-rank *p*-value for differences between curves; (**C**) funnel plot for Egger test to assess evidence of bias due to small study effect.

We observed significantly more favorable OS among patients with HPV+ SNSCC, with a summary HR = 0.51 (95% CI: 0.38–0.70; Figure 2A). This was also reflected in the summary survival curves (Figure 2B), which showed significantly better 5-year OS among patients with HPV+ SNSCC (67.6%), compared to those with HPV- SNSCC (47.6%; p_log-rank_ = 3.62 × 10^−7^). Heterogeneity was relatively low between the 11 studies reporting OS by HPV-status (*p* = 0.34, I^2^ = 11.24%). There was no evidence of bias due to the small study effect (*p* = 0.97; Figure 2C).

Although the differences between subgroups were not statistically significant (*p* = 0.49), it should be noted that studies using HPV E6/E7 mRNA had a lower meta HR than those using HPV DNA-based testing. This suggests the need for future studies assessing the degree of concordance and survival comparisons between HPV detection methods for SNSCC. 

We additionally conducted several post hoc sensitivity analyses to test the robustness of our meta-estimates of OS (Supplemental Figures S1–S3). These included the exclusion of the heaviest weighted study (Oliver et al. [24]), exclusion of studies that necessitated indirect HR estimates, and use of random effects (as opposed to the fixed effects model that was reported), resulting in comparable summary HRs of 0.56 (95% CI: 0.38–0.85), 0.55 (95% CI: 0.29–1.04), and 0.52 (95% CI: 0.36–0.74), respectively.

### 2.5. Meta-Analysis of Disease-Free Survival

**Figure 3:**
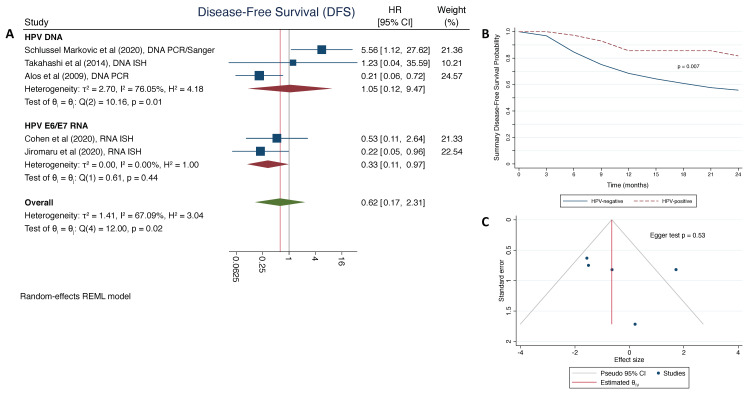
Summary estimates for the association between human papillomavirus (HPV)-status and disease-free survival (DFS) for patients with sinonasal squamous cell carcinoma (SNSCC): (**A**) forest plot of overall survival meta-analysis with summary hazard ration (HR) stratified by HPV detection method; (**B**) summary Kaplan-Meier curve for 2-year DFS of SNSCC patients by HPV status with log-rank p-value for differences between curves; (**C**) funnel plot for Egger test to assess evidence bias due to small study effect.

We did not observe a significant association between HPV-status and DFS (Figure 3A), with a summary HR = 0.62 (95% CI: 0.17–2.31). However, this may be a reflection of the comparatively limited statistical power with a relatively high degree of heterogeneity between the four studies reporting DFS by HPV-status (*p* = 0.01, I^2^ = 67.%), in large part driven by the Schlussel Markovic study [25]. It is notable that the summary HR point estimate for HPV+ SNSCC is comparable to that observed for OS and, further, the summary survival curves (Figure 3B) showed significantly better 2-year DFS among patients with HPV+ SNSCC (81.7%), compared to those with HPV- SNSCC (55.8%; p_log-rank_ = 0.007). No significant differences were observed by HPV assay type (*p* = 0.27), although the number of studies per subgroup was limited. There was no evidence bias from the small study effect (*p* = 0.53; Figure 3C).

## 3. Discussion

This meta-analysis provides summary evidence of favorable survival outcomes associated with HPV+ SNSCC, in terms of overall survival (OS), and provides limited evidence for a potential association with disease-free survival (DFS), although statistical power was less for the latter due to the smaller number of studies reporting on DFS. These findings are notable in that the observed association is in line with that reported for OPSCC [29]. While HPV is widely accepted as a prognostic factor for OPSCC, the association with survival at other non-oropharyngeal head and neck subsites (e.g., oral cavity, larynx, hypopharynx and nasopharynx) has been much less clear with mixed results [5,29,30,31,32,33,34,35,36].

Understanding the role of HPV in head and neck cancer is an active and important area of research [9]. As the incidence of HPV-driven head and neck cancers continues to rise [37], determining the optimal management of these patients and whether these patients should be treated similarly or differently than their HPV- counterparts are presently open questions. Despite disappointing results from the RTOG-1016 (NCT01302834) [38] and De-ESCALaTE HPV (ISRCTN33522080) trials [39], which demonstrated poorer overall and disease-free survival in patients treated with radiotherapy plus cetuximab, rather than standard-of-care radiotherapy plus cisplatin, this may be viewed as more of a contraindication for substitution or elimination of cisplatin [9]. Notwithstanding, there is still substantial interest in exploring treatment de-escalation for HPV+ head and neck squamous cell carcinoma using other strategies [40], and this remains an active area of research. For example, studies such as NRG-HN002 (NCT02254278) have already identified a subpopulation of patients in which reduced doses of radiation may be as effective as conventional doses [41]. As sinonasal cancers often present at advanced stage, with cranial nerve deficits or close abutment to critical structures, including the orbit and optic nerves, a surgical approach has been historically preferred. However, newer studies including ECOG-ACRIN 3163 (NCT03493425) question whether neoadjuvant chemotherapy to reduce the tumor size may allow for less morbid surgery [42]. As there is limited data on HPV and SNSCC prognosis, this trial does not stratify by HPV-status, but as more information becomes available, such as we have presented here, the possibility of stratifying by SNSCC by HPV-status to determine whether or not a patient could achieve adequate disease control with radiation and/or chemotherapy versus surgery will need to be addressed.

A major strength of this meta-analysis stems from the inherent aggregate nature of meta-analysis that allowed for increased statistical power to assess the association between HPV and survival, which, given the relative rarity of SNSCC, allowed us to draw conclusions that may not have otherwise been possible. Other strengths include the low heterogeneity between studies reporting OS, and the absence of evidence of bias from a small study effect. However, there are also some limitations. Not all studies provided HRs, necessitating indirect estimations from the Kaplan-Meier curves, which are unadjusted for potential confounding factors and may not precisely align with the true measured effect. Additionally, many of the studies were comparatively small in size due to the relative rarity of SNSCC, limiting their precision and statistical power. However, there was no evidence of a small study effect suggesting limited potential for bias, and, as stated above, a strength of meta-analysis is the ability to increase statistical power by combining estimates across studies. Studies also reported HPV using a variety of methods in varying study populations, but heterogeneity between studies assessing OS was relatively low and differences in summary estimates between HPV assay subgroups were non-significant, suggesting that this was not a major factor. Lastly, a limitation of meta-analysis in general is that summary estimates are based on aggregate results from each publication, rather than individual-level patient data, which could potentially introduce bias, particularly for studies that did not provide adjusted hazard ratio(s). However, it is notable that heterogeneity between studies reporting OS, including both those that provided adjusted HR(s) and those that did not, was very low, which seems to suggest a limited impact of indirect estimation.

## 4. Methods

### 4.1. Study Identification and Selection

Studies reporting on the association between HPV-status and SNSCC survival outcomes were identified via PubMed using the following search terms: *(hpv OR papillomavirus) AND (sinonasal OR sinus OR nasal) AND (cancer OR carcinoma) AND (prognosis OR survival OR outcome)*. Search results were independently screened by 2 of the authors (AS and SML). For inclusion, studies had to be original research studies published in the English language through 1 February 2021 that reported OS or DFS for SNSCC by HPV-status using a definitive HPV test; studies reporting survival by p16 immunohistochemistry only were excluded. Post hoc searches of Scopus and Web of Science databases were additionally conducted, and references of all relevant studies were crosschecked for any studies that may have been missed by the initial search. If multiple studies were identified with overlapping study populations, the most inclusive was retained. This systematic review and meta-analysis were conducted and written in accordance with the Preferred Reporting Items for Systematic reviews and Meta-Analyses (PRISMA) [43] and registered with the PROSPERO international database of prospectively registered systematic reviews (pending) [44].

### 4.2. Data Extraction

Potential eligibility was preliminarily assessed by screening the abstracts of articles produced from the PubMed search. The full text of articles that were not excluded during the screening process was further scrutinized to determine eligibility. Descriptive study characteristics were extracted from all eligible articles, including study year, country, years of diagnosis for the initial primary tumors, HPV assay, number/frequency of HPV+ and HPV- cases, primary survival outcome(s), maximum follow-up time, percent of male cases, and median age at diagnosis (Table 1). HR for OS and/or DFS for HPV+ versus HPV- SNSCC and a corresponding 95% confidence intervals (CI) or data allowing for indirect estimation, as described below, were extracted from the full text.

### 4.3. Qualitative Assessment of Study Quality and Potential Risk for Bias

Study quality and risk of bias were qualitatively assessed based on 6 investigator-defined items using the criteria described in Table 2, with an overall score assigned based on holistic consideration of all items. Scores were assigned as (A) high-quality with low risk for bias; (B) moderate quality and risk for bias; or (C) low quality and/or high risk for bias. For sample size, quality scores were assessed based on the following criteria: (A) >100 cases and >20 HPV+ cases; (B) >50 cases but <100 cases; (C) <50 cases or <10 HPV+ cases.

### 4.4. Summary Survival Curve Estimation

Summary survival curves for 5-year OS and 2-year DFS were estimated by systematically parsing each study-specific Kaplan-Meier survival curve into equal, prespecified, non-overlapping time intervals (6-month intervals for OS; 3-month intervals for DFS) and estimating survival probability (Si) for HPV+ and HPV- cases, respectively, at each interval. Censoring was assumed to be noninformative and occurring at a constant rate. The number of patients censored at each time interval, C*_i_*(t*_i_*), was estimated by $C_{i}\left(t_{i}\right)=\frac{R_{i}\left(t_{\mathrm{si}}\right)*\left(t_{\mathrm{ei}}-t_{\mathrm{si}}\right)}{2*\left(F_{\max}-t_{\mathrm{si}}\right)}$, where R*_i_* is the number at-risk, t_s_ is the start of the interval, t_e_ is the end of the interval, and F_max_ is the maximum follow-up in the study [45]. At-risk patients during each interval were calculated as R*_i_*(t) = R*_i_*(t_s_) − C*_i_*(t). Deaths for each time interval were calculated as $D_{i}\left(t_{i}\right)=R_{i}\left(t\right)*\frac{Si\left(t-1\right)-Si\left(t\right)}{Si\left(t-1\right)}$. Summary survival curves were then generated by HPV-status using the actuarial method based on aggregate at-risk cases, censored cases and deaths for each time interval. Differences between summary curves were formally evaluated via log-rank test [46].

### 4.5. Meta-Estimate of Hazard Ratio

HR and a corresponding 95% CI for the association of HPV-status with OS and DFS were extracted from each study. If the study did not provide HR, it was indirectly estimated by $\ln\left(\mathrm{HR}\right)=\frac{\left(O_{pos}-E_{pos}\right)}{\hat{V}}=\frac{\sqrt{{O*R}_{neg}{*R}_{pos}}{*\Phi }^{-1}\left(1-\frac{p}{2}\right)}{\frac{{O*R}_{neg}{*R}_{pos}}{R_{neg}+R_{pos}}}$ [45], where O is the total number of events between both HPV-positive and HPV-negative cases, O*_pos_* and E*_pos_* represent the respective observed and expected events for HPV-positive SNSCC patients, $\frac{1}{V}$ is the estimated Mantel–Haenszel variance of the log-HR, R*_neg_*, and R*_pos_* are the respective number of at-risk HPV+ and HPV- cases, *p* is the two-sided log-rank *p*-value for a survival difference by HPV-status, and Φ is the cumulative-distribution function for a standard normal distribution. Alternatively, when insufficient data were provided for the indirect estimation described above, HR and variance was estimated from the Kaplan-Meier function using the following formulas, where D*_pos_*(*t*) and D*_neg_*(*t*) and R*_pos_*(*t*) and R*_neg_*(*t*) are estimated deaths and number of at-risk cases at each prespecified time interval for HPV+ and HPV- SNSCC, respectively:

$$\ln(\mathrm{HR})=\frac{\sum_{t=1}^{T}\frac{\ln\left(\frac{D_{pos}\left(t\right)/R_{pos}\left(t\right)}{D_{neg}\left(t\right)/R_{neg}\left(t\right)}\right)}{\left(\frac{1}{D_{pos}\left(t\right)}-\frac{1}{R_{pos}\left(t\right)}+\frac{1}{D_{neg}\left(t\right)}-\frac{1}{R_{neg}\left(t\right)}\right)}}{\sum_{t=1}^{T}\frac{1}{\left(\frac{1}{D_{pos}\left(t\right)}-\frac{1}{R_{pos}\left(t\right)}+\frac{1}{D_{neg}\left(t\right)}-\frac{1}{R_{neg}\left(t\right)}\right)}}\;\mathrm{and}\;\mathrm{var}\left[\ln\left(\mathrm{HR}\right)\right]={\left[\sum_{t=1}^{T}\left(\frac{1}{D_{pos}\left(t\right)}-\frac{1}{R_{pos}\left(t\right)}+\frac{1}{D_{neg}\left(t\right)}-\frac{1}{R_{neg}\left(t\right)}\right)\right]}^{-1}\;$$

Meta-analyses were conducted for the association of HPV-status with OS and DFS, stratified by the HPV detection method (*HPV DNA*, *HPV E6/E7 mRNA*, or *unspecified*). Summary HR was generated via a fixed-effects model (inverse variance method) [47] and random-effects model using the restricted maximum likelihood (REML) heterogeneity variance estimator [48]. Between-study heterogeneity was quantitatively assessed using the Q-statistic and I^2^ metric [49]. When heterogeneity was low (Q-statistic *p* > 0.05 and I^2^ < 30%), fixed-effect estimates were reported to conserve statistical power; otherwise random-effects were reported. The risk of small study effect (“publication bias”) across studies was assessed using the Egger test [50].

Analyses were conducted using Stata/16 (StataCorp LLC, College Station, TX, USA).

## 5. Conclusions

The findings of this systematic review and meta-analysis indicate that HPV is a significant predictor of more favorable survival for SNSCC patients and suggest that HPV may be a useful biomarker for prognostication. It also highlights the need for additional, well-designed and adequately powered studies to provide better precision and additional insight into the relationship between HPV and SNSCC outcomes and variability among HPV testing modalities.

## Figures and Tables

**Table 1:** Characteristics of studies included in the meta-analyses.

Study	Year Published	Country	Years of Diagnosis	HPV Detection Method	HPV-Positive, *n* (%)	HPV-Negative, *n* (%)	Primary Outcomes	Median Follow-Up (Months)	Maximum Follow-Up (Months)	Percent Male	Median Age (Years)
Schlussel Markovic et al.	2020	USA	2011–2018	DNA PCRSanger	17 (53%)	15 (47%)	OS, DFS ^b^	30.7	>60	66%	58
Cohen et al.	2020	USA	2011–2017	RNA ISH	10 (45%)	12 (55%)	OS, DFS	DNR	>60	55%	DNR
Jiromaru et al. ^a^	2020	Japan	2003–2016	RNA ISH	9 (9%)	92 (91%)	OS, DFS	DNR	>60	69%	64 ^c^
Alos et al.	2009	Spain	1981–2006	DNA PCR	12 (20%)	48 (80%)	OS, DFS	24	>60	75%	HPV+ = 69 HPV- = 62
Takahashi et al.	2014	USA	1999–2009	DNA ISH	6 (9%)	58 (91%)	OS, DFS	33.9	60	50%	DNR
Hongo et al.	2021	Japan	2003–2019	RNA ISH	11 (8%)	126 (92%)	OS	DNR	>60	70%	64.4 ^c^
Svajdler et al.	2020	Czech Republic, Slovakia	2002–2014	RNA ISH RT-PCR	8 (24%)	26 (76%)	OS	23.3	>60	71%	HPV+ = 56 HPV- = 58
Oliver et al.	2019	USA	2010–2016	Unspecified	128 (34%)	254 (66%)	OS	24	48	65%	64 ^d^
Chowdhury et al.	2017	USA	1990–2015	DNA PCR	16 (62%)	10 (38%)	OS	DNR	>60	73%	64.5
Laco et al.	2015	Czech Republic	1995–2014	DNA ISH DNA PCR RNA ISH RT-PCR	17 (35%)	32 (65%)	OS	16	>60	69%	65
Bishop et al.	2013	USA	1995–2011	DNA ISH	20 (29%)	49 (71%)	OS	DNR	>60	DNR	DNR
Furuta et al.	1992	Japan	1979–1988	DNA-PCR	7 (14%)	42 (86%)	OS	DNR	60	69%	57.3

^a^ Included in meta-analysis of DFS only due to overlap with Hongo et al. ^b^ Only included local recurrence; distant metastasis/recurrence was assessed separately. ^c^ Mean age (years). ^d^ Based on all sinonasal squamous cell carcinoma cases in the National Cancer Database. USA = United States of America; ISH = in situ hybridization; PCR = polymerase chain reaction; RT-PCR = reverse-transcriptase polymerase chain reaction; HPV = human papillomavirus; OS = overall survival; DFS = disease-free survival; DNR = did not report.

**Table 2:** Qualitative assessment of quality and potential risk of bias for the studies included in the meta-analysis.

Item	Criteria	Quality Assessment ^a^											
		Schlussel Markovic	Cohen	Jiromaru	Alos	Hongo	Svajdler	Oliver	Chowdury	Laco	Bishop	Furuta	Takahashi
Sample size	Was the sample size adequate for drawing meaningful conclusions, including a sufficient number of HPV-positive cases?	C	C	C	B	B	C	A	C	C	B	C	B
Case selection	Were cases representative of the general sinonasal squamous cell carcinoma (SNSCC) patient population? Was criteria for inclusion/exclusion clearly described? Were clinical and demographic characteristics adequately reported?	B	A	B	B	B	B	A	B	A	B	B	B
Kaplan-Meier curves	Did the investigators provide Kaplan-Meier survival curves and report an appropriate statistical comparsion, such as log-rank test?	A	A	A	A	A	A	A	A	A	A	B	C
Confounding factors	Did the investigators adjust for potential confounding factors, such as age, sex, race/ethnicity and/or clinical factors?	C	C	B	B	B	C	A	C	C	A	C	C
Survival model assumptions	Did the investigators test for underlying model assumptions, where applicable, such as proportional hazards?	n/a	n/a	C	C	C	C	C	n/a	n/a	C	n/a	n/a
Human papillomavirus (HPV) assay	Was the type of HPV assay reported? Did the assay test for an appropriate spectrum of high-risk HPV types? Was criteria for HPV positivity clearly defined? If in-situ hybridization was used, were the slides interpreted by multiple blinded observers?	A	B	B	A	B	B	C	A	A	B	C	B
**Overall study quality**	Overall assessment of study quality based on the six investigator-defined criteria listed above	C	C	C	B	B	C	B	C	C	B	C	C

^a^ Quality assessment scores; A = high quality/low risk for bias; B = moderate quality/moderate risk for bias; C = low quality/high risk for bias; n/a = not applicable.

## Supplementary Materials

The following are available online at https://www.mdpi.com/article/10.3390/cancers13153677/s1, Figure S1: Forest plot for the sensitivity analysis for the association of human papillomavirus and overall survival for sinonasal squamous cell carcinoma excluding of the heaviest weighted study; Figure S2: Forest plot for the sensitivity analysis for the association of human papillomavirus and overall survival for sinonasal squamous cell carcinoma excluding the six studies requiring indirect estimation of hazard ratio; Figure S3: Forest plot for the sensitivity analysis for the association of human papillomavirus and overall survival for sinonasal squamous cell carcinoma using a restricted maximum likelihood random-effects model.
