# Supplementary material for: Human Papillomavirus and Survival of Sinonasal Squamous Cell Carcinoma Patients: A Systematic Review and Meta-Analysis
Source: Cancers (Basel). 2021 Jul 22;13(15):3677. doi: 10.3390/cancers13153677 (PMC8345036; doi:10.3390/cancers13153677)
Supplement: Supplementary file 1 [file cancers-13-03677-s001.zip › cancers-1262616-supplementary.pdf]

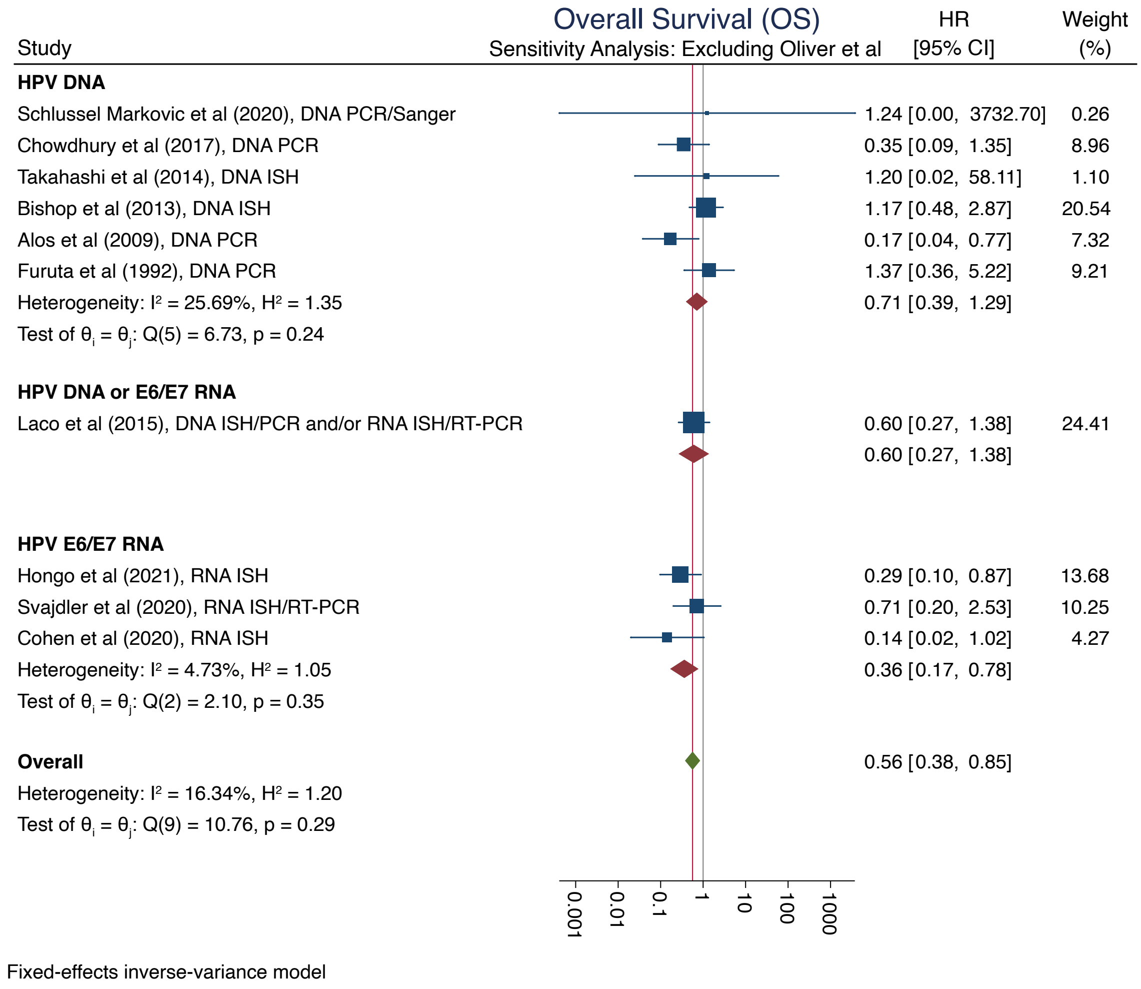

Supplemental Figure S1. Forest plot for the sensitivity analysis for the association of human papillomavirus (HPV) and overall survival (OS) for sinonasal squamous cell carcinoma (SNSCC) excluding of the heaviest weighted study (Oliver et al, 2020).

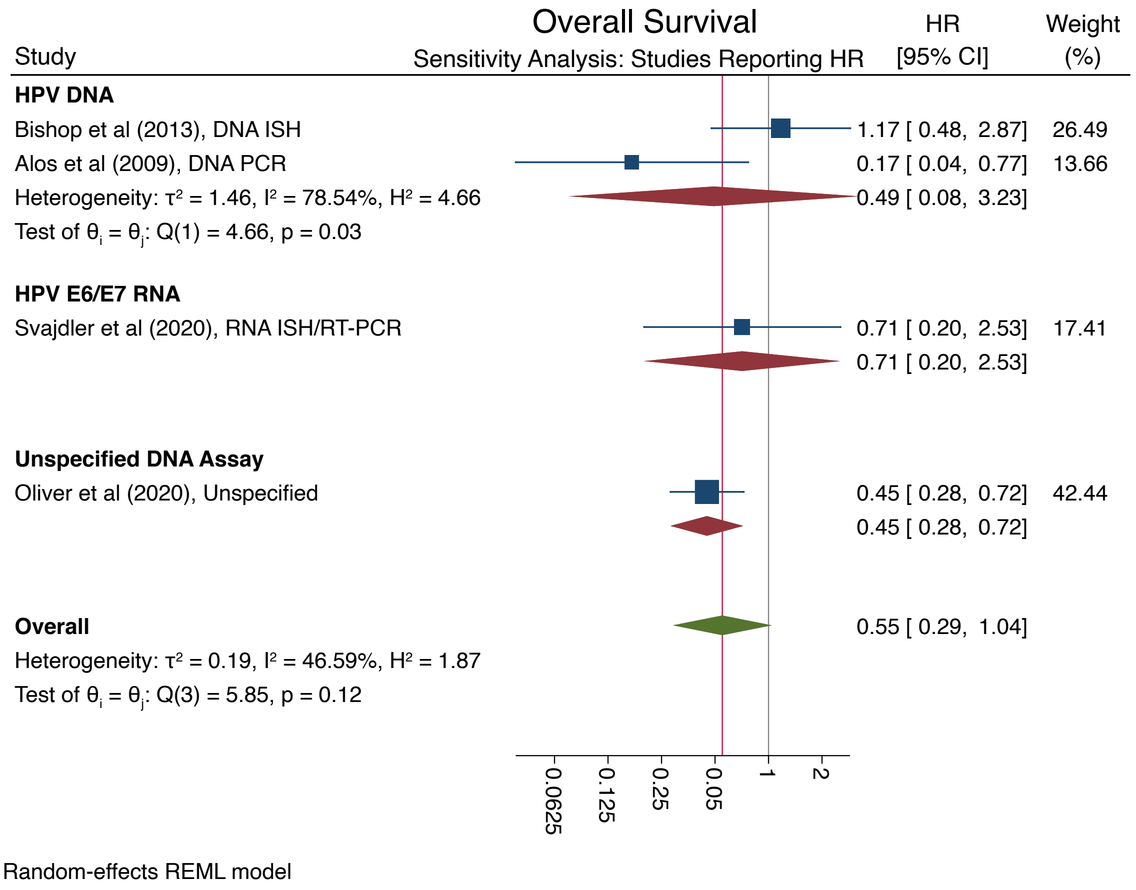

Supplemental Figure S2. Forest plot for the sensitivity analysis for the association of human papillomavirus (HPV) and overall survival (OS) for sinonasal squamous cell carcinoma (SNSCC) excluding the six studies requiring indirect estimation of hazard ratio (HR).

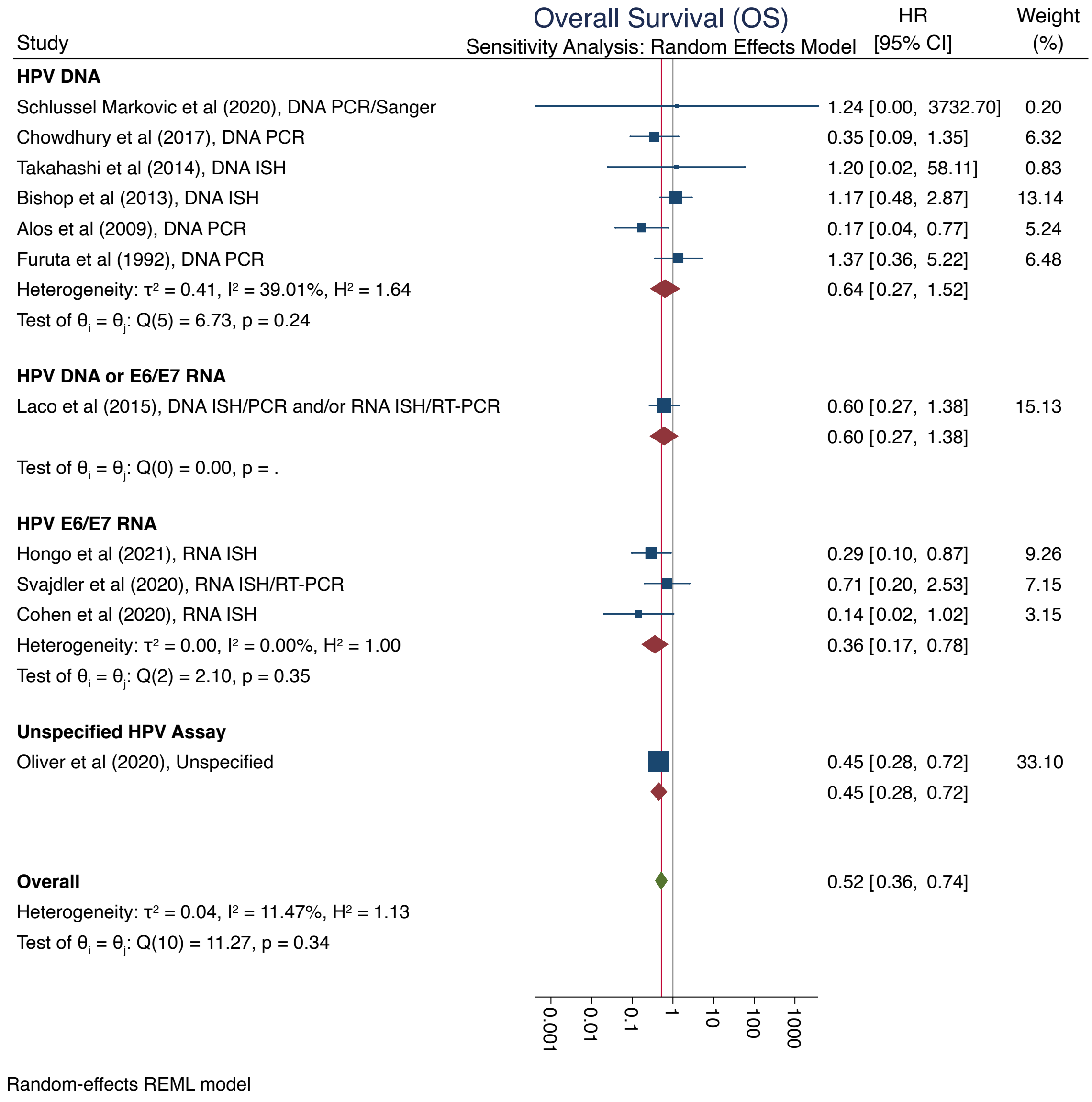

Supplemental Figure S3. Forest plot for the sensitivity analysis for the association of human papillomavirus (HPV) and overall survival (OS) for sinonasal squamous cell carcinoma (SNSCC) using a restricted maximum likelihood random-effects model (REML).
